# Case Report: Cardiac angiosarcoma with rib pain as the first symptom

**DOI:** 10.3389/fonc.2026.1815522

**Published:** 2026-04-13

**Authors:** Meng Cai, Xiaodi Chen, Jie Li, Rong Liu, Chang Zhou

**Affiliations:** Department of Ultrasound Imaging, The First College of Clinical Medical Science, China Three Gorges University, Yichang Central People’s Hospital, Yichang, China

**Keywords:** bone metastases, cancer pain, case report, primary cardiac angiosarcoma, rib pain

## Abstract

Primary cardiac angiosarcoma (PCAS) is a rare, highly aggressive malignancy characterized by a dismal prognosis. We present a case of a 34-year-old woman with no history of trauma who initially presented with persistent rib pain misdiagnosed as costochondritis. Comprehensive evaluation revealed multiple osteolytic bone metastases originating from a right atrial PCAS, accompanied by circumferential pericardial effusion. Despite initiation of palliative chemotherapy and radiotherapy, the patient succumbed to progressive disease five months after diagnosis. This case underscores three critical clinical considerations: (1) metastatic bone involvement may manifest solely as localized, refractory pain; (2) isolated pain without overt cardiac symptoms poses significant diagnostic challenges and risks delayed recognition; and (3) unexplained bone pain—particularly when concurrent with pericardial effusion—warrants prompt multimodal imaging and interdisciplinary evaluation involving oncology, cardiology, and pain medicine. By detailing the clinical, radiological, and histopathological findings, we aim to raise awareness among pain specialists and oncologists of this rare yet life-threatening cardiac malignancy and to advocate earlier diagnostic suspicion in atypical pain presentations.

## Introduction

Pain is a common, profoundly debilitating, and psychologically distressing symptom that persists for many individuals, including cancer survivors. Cancer pain is a predominant symptom among patients with malignant diseases, occurring in 49.2% of patients during treatment and in 40.1% after treatment (1, 2). It arises primarily from tumor-related mechanisms (e.g., compression, ischemia) or treatment toxicities (e.g., surgery, chemotherapy, or radiation) (3). The prevalence of self-reported pain varies significantly across malignancies: lung cancer has the highest incidence among primary tumors, followed by gastric and hepatocellular carcinomas (4); in metastatic disease, bone metastases are the predominant pain source, exceeding hepatic and cerebral metastases in clinical impact (4, 5). Notably, bone metastases from common primaries (e.g. breast, prostate, or lung) are well-established major contributors to cancer-related pain (5).

In stark contrast, primary cardiac tumors presenting with bone pain as the inaugural symptom are exceptionally rare. With an annual incidence of only 1.38 per 100,000 individuals—and malignant subtypes constituting merely 9.5% of cases (6)—cardiac malignant origins are seldom considered in the differential diagnosis of unexplained skeletal pain. Consequently, clinicians routinely prioritize orthopedic, rheumatologic, or common metastatic etiologies, inadvertently overlooking the heart as a potential source. This diagnostic blind spot may delay critical intervention in highly aggressive cardiac malignancies.

Herein, we report a unique case of a 34-year-old woman who presented with intractable rib pain as the sole initial symptom. Comprehensive evaluation revealed multiple osteolytic bone lesions and pericardial effusion, ultimately diagnosed as metastatic disease originating from a primary cardiac angiosarcoma of the right atrium.

## Case description

A 34-year-old woman presented to the Pain Management Clinic at a local county hospital with a 2-month history of persistent left 2nd to 4th rib pain adjacent to the sternum. With no significant medical history and a recent normal physical examination (**Supplementary Data**), an initial diagnosis of costochondritis was made by Pain Department, and she underwent common intercostal nerve block therapy. However, the analgesic effect was unsatisfactory, lasting only 3 days, and she was referred to the Orthopedics Department. Contrast-enhanced chest computed tomography (CT) revealed osteolytic destruction of the left 6th and 7th ribs with adjacent soft tissue masses and a circumferential pericardial effusion. Given the highly concerning co-occurrence of lytic rib lesions and pericardial effusion—two red flags for systemic malignancy—she was urgently transferred to our tertiary referral center for comprehensive evaluation.

**Figure 1:**
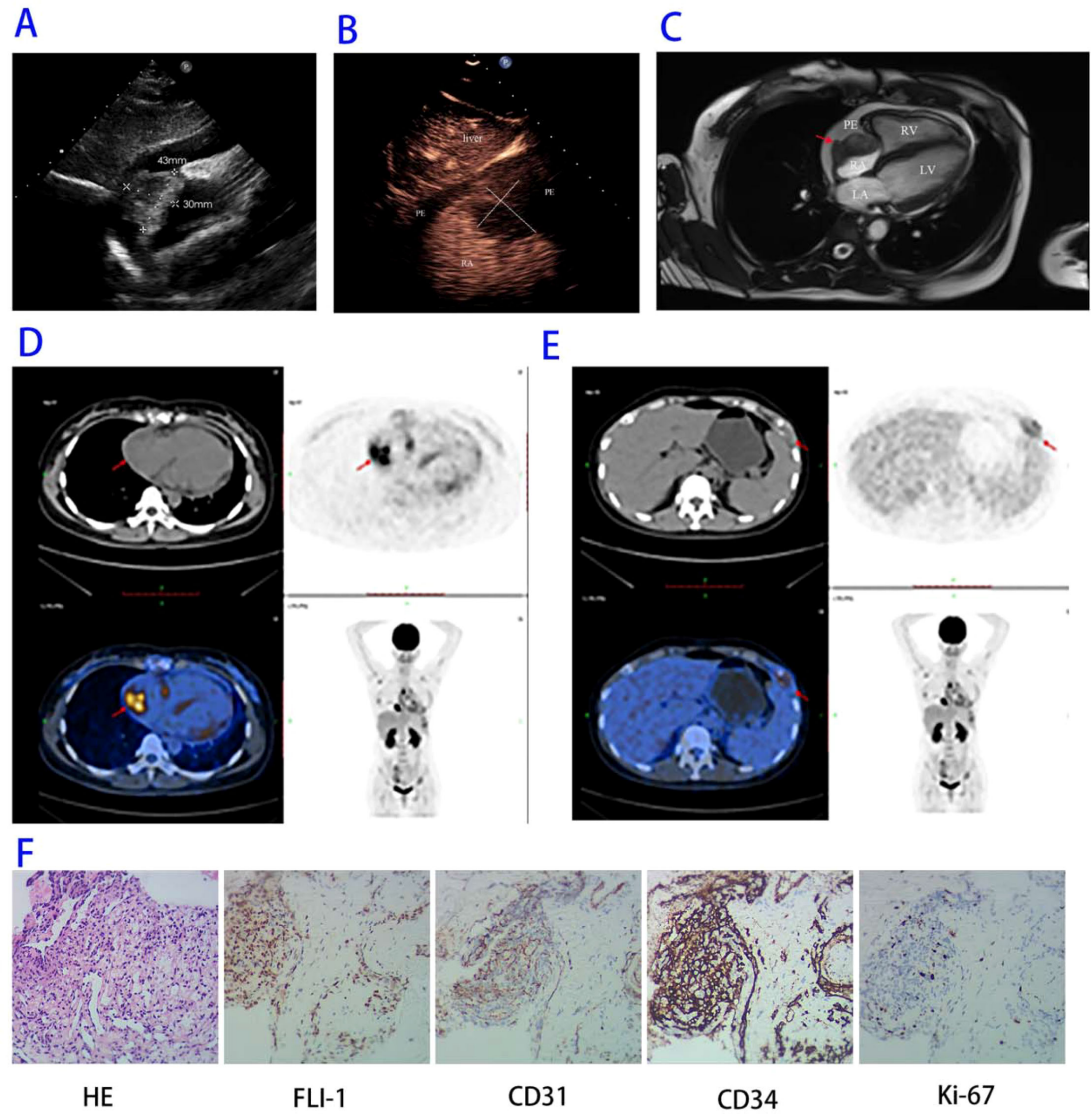
**(A)** Transthoracic echocardiography in the subcostal four-chamber view showing a lobulated, iso-echoic mass (measuring 43 × 30 mm, as indicated by calipers) originating from the right atrial free wall, with extension into both the right atrial cavity and pericardial cavity , accompanied by pericardial effusion. **(B)** Contrast-enhanced echocardiography in the subcostal two-chamber view revealing a filling defect within the right atrium (calipers), with slight contrast enhancement within the mass. **(C)** Cardiac MRI delineating the mass (arrow) originating from the right atrial free wall, exhibiting a lobulated morphology with slightly longer T1 and T2 signal intensity, accompanied by a circumferential pericardial effusion. **(D, E)** PET-CT images depicting intense high metabolic activity at the right atrial mass [arrow, Figure Panel **(D)**] and left rib lesions [arrow, Panel **(E)**]. **(F)** Histopathology (H&E, ×20) revealing epithelioid endothelial cells with mild-to-moderate atypia; Immunohistochemistry staining positive for FLI-1, CD31, CD34, and Ki-67 LI: 20%.

Transthoracic echocardiography identified a lobulated, moderately echogenic mass (43 × 30 mm) arising from the right atrial free wall, extending into both the right atrial cavity and pericardial cavity with ill-defined borders involving the distal superior vena cava (**Figure 1A**; **Supplementary Video 1**). Circumferential pericardial effusion was noted, measuring 10 mm at the LV inferior wall, 5 mm at the RV anterior wall, 7 mm at the LV lateral wall, 9 mm at the RV lateral wall, and 8 mm at the roof of the right atrium (**Supplementary Videos 2** and **3**). Contrast-enhanced echocardiography demonstrated a filling defect within the right atrium, with slight contrast perfusion in the mass (**Figure 1B**; **Supplementary Video 4**). Cardiac Magnetic Resonance Imaging (MRI) delineated the mass originated from the right atrial free wall, exhibiting a lobulated morphology with slightly longer T1 and T2 signal intensity (**Figure 1C**). Positron Emission Tomography–Computed Tomography (PET-CT) demonstrated intense 18-fluorodeoxyglucose (18F-FDG) avidity at the right atrial lesion (SUVmax elevated), confirming high metabolic activity consistent with malignancy (**Figure 1D**). Concurrently, multiple hypermetabolic osteolytic lesions were identified in the sternum, ribs, and sacrum, establishing widespread skeletal metastases (**Figure 1E**).

Histopathological analysis of a sternal biopsy revealed sheets of epithelioid endothelial cells with mild-to-moderate nuclear atypia (H&E, ×20). Immunohistochemistry was positive for vascular endothelial markers Friend Leukemia Integration 1(FLI-1), Cluster of Differentiation 31(CD31), and Cluster of Differentiation 34 (CD34), with a Ki-67 labeling index of 20% (**Figure 1F**), confirming the diagnosis of angiosarcoma.

Following confirmation of stage IV right atrial primary angiosarcoma with widespread osseous metastases, the multidisciplinary team (MDT) determined that curative resection was precluded by extensive metastatic burden and local invasiveness. A palliative systemic therapy regimen was initiated: liposomal doxorubicin (40 mg, day 1) combined with ifosfamide (2.9 g/day, days 1–5), mensa (600 mg at 0, 3, 6, and 9 hours post-ifosfamide), and bevacizumab (200 mg, day 1). However, during the first night after treatment initiation, the patient developed severe dyspnea and intense vomiting, necessitating discontinuation of chemotherapy. Subsequently, she received palliative radiotherapy targeting the cardiac and bone metastatic lesions (95% planning target volume [PTV] 50 Gy delivered in 25 fractions of 2 Gy each), and together with symptomatic supportive care including analgesia and leukocyte/platelet support. Simultaneously, zoledronic acid was administered for the management of bone metastases. Approximately two months post-radiotherapy initiation, the patient developed new-onset perioral numbness accompanied by left-sided facial pain that worsened at night. The pain was poorly controlled with sustained−release oxycodone and pregabalin. Following MDT reassessment, a diagnostic trigeminal nerve block provided marked analgesic response, confirming neuropathic involvement. Despite aggressive supportive interventions, progressive disease led to death 5 months after diagnosis.

## Discussion

Primary cardiac angiosarcoma (PCAS) is a rare malignant tumor arising from the endothelial cells of cardiac blood vessels, representing approximately 25%–30% of all primary cardiac tumors (7). Predominantly located in the right atrium, PCAS often presents insidiously with nonspecific cardiac symptoms-such as palpitations, chest pain, dyspnea, or cough-due to local tumor enlargement or pericardial effusion (8–12). While distant metastases (most commonly to the lungs, liver, and lymph nodes) may manifest with extracardiac symptoms such as hemoptysis, pulmonary infection, or neurological deficits, or acute brain hemorrhage (13–15), a growing body of case reports evidence highlights localized pain as an initial and dominant symptom, frequently leading to misdiagnosis.

In our case, persistent rib pain—initially attributed to costochondritis—was later identified as osteolytic destruction caused by bone metastases. This aligns with multiple reported cases: spinal destruction causing back pain (16); pleuritic chest pain from a large right atrial mass (17); pericarditis-mimicking chest pain (18); mediastinal compression-induced abdominal pain (19); and acute epigastric pain due to right atrial rupture (20). These observations underscore that PCAS-related pain may originate not only from hematogenous bone metastases but also from direct invasion through the fibrous pericardium into adjacent ribs, chest wall or nerve compression syndromes. In our patient, neuropathic pain intensified progressively after each treatment cycle, necessitating multimodal analgesia (morphine, pregabalin, oxycodone).

Pericardial effusion in malignant tumor patients has multifactorial etiologies: it may stem from direct tumor invasion or metastasis to the pericardium via lymphatic or hematogenous routes, arise from inflammatory processes (e.g., opportunistic infections), or result from radiotherapy or chemotherapy-related toxicity (21). Management requires individualized assessment based on effusion volume, clinical presentation, and overall prognosis. For small or localized effusions, conservative observation is recommended to avoid unnecessary invasive procedures; however, when progressive accumulation leads to ventricular compression and hemodynamic instability, prompt pericardiocentesis is essential to alleviate symptoms and prevent cardiogenic shock. In our case, tumor pathology was confirmed by sternal biopsy. Although pericardial effusion persisted, the volume fluctuated between small and moderate without significantly impacting cardiac function, so no specific intervention was performed. Notably, pericardial effusion is a hallmark of advanced malignancy, and recurrent effusion is a primary and common challenge—even in patients with PCAS. Multiple case studies indicate that PCAS-related effusions are typically exudative with negative cytology, limiting their diagnostic specificity (22–26). Pericardiocentesis alone provides inadequate long-term palliation; thus, long-term management requires a multimodal approach, including balloon pericardiotomy, systemic antitumor therapy, or localized interventions (21).Fortunately, during the treatment period, our patient’s pericardial effusion did not pose a threat to cardiac function, and no radiotherapy- or chemotherapy-related toxic pericarditis occurred. When unexplained localized pain (e.g., rib, back, or abdominal) coexists with pericardial effusion, PCAS should be strongly considered. Our case exemplifies the diagnostic pitfall of attributing such pain to benign musculoskeletal conditions. Early multimodal imaging (echocardiography, cardiac MRI, or contrast-enhanced CT) coupled with interdisciplinary evaluation is critical to avoid delays in diagnosis.

No standardized treatment protocol exists for PCAS due to the scarcity of high-level evidence; thus, management remains highly individualized based on disease extent. According to the study by Basel Ramlawi et al.—the largest single-center surgical series of PCAS—mean survival without resection was only four to six months, whereas median survival reached 27 months with resection, with a 5-year survival rate of approximately 36% (27). Given a low R0 resection rate (38%) and a high incidence of metastatic disease at presentation (>80%), the large surgical cohort of Basel Ramlawi et al. advocates for a sequential therapeutic strategy (27). A biopsy for histopathological diagnosis was first followed by neoadjuvant chemotherapy. Surgical intervention is selectively reserved for patients demonstrating a favorable response to systemic therapy. Specifically, patients with metastatic disease who achieve a complete or near-complete systemic response, in conjunction with a substantial local tumor response, are considered surgical candidates. Conversely, patients exhibiting inadequate local or metastatic response to chemotherapy are deemed unresectable and continue to receive systemic chemotherapy as definitive management, with palliative therapy considered on a case-by-case basis. In brief, neoadjuvant chemotherapy is used to identify true surgical candidates, thereby sparing patients with extensive metastasis or chemorefractory disease from high-risk, low-yield operations. Our patient’s clinical trajectory further underscores the therapeutic challenges in advanced PCAS: profound treatment intolerance limits systemic options, necessitating a timely integration of localized therapies and precision symptom control to preserve quality of life. This case highlights a critical unmet need in PCAS management—the development of innovative therapeutic strategies that concurrently target tumor biology and underlying pain pathways, integrating precision oncology with mechanism-based analgesia to address both disease progression and symptom burden.

## Conclusion

PCAS should be included in the differential diagnosis of unexplained localized pain—particularly when associated with pericardial effusion. Heightened clinical suspicion, timely advanced imaging, and multidisciplinary collaboration are essential to improve diagnostic accuracy and patient outcomes in this aggressive malignancy.

## Data Availability

The original contributions presented in the study are included in the article/**Supplementary Material**. Further inquiries can be directed to the corresponding authors.
