# Supplementary material for: Case Report: Cardiac angiosarcoma with rib pain as the first symptom
Source: Front Oncol. 2026 Apr 13;16:1815522. doi: 10.3389/fonc.2026.1815522 (PMC13111074; doi:10.3389/fonc.2026.1815522)
Supplement: Supplementary file 1 [file SupplementaryFile1.zip › video files/supplementary data.docx]

These were the physical examination reports from the three months prior to the patient's initial visit to the Pain Department, as follows:

**Physical Examination 1**​. **Comprehensive Tumor Marker Panel for Women**

**
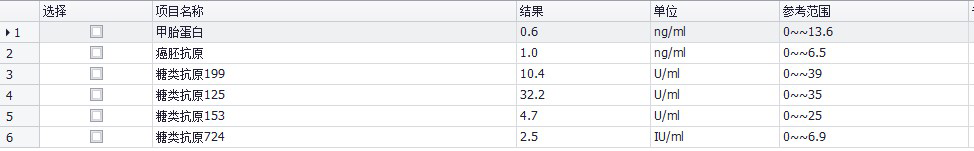
**

**Physical Examination 2**​. **Serum Immunofixation Electrophoresis**

**
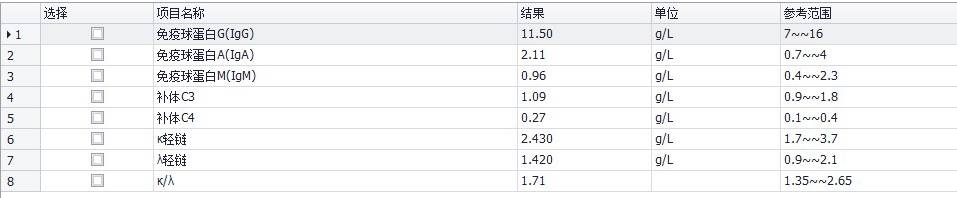
**

**Physical Examination 3**​. **Thyroid Function**
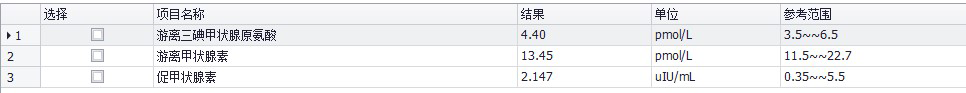


**Physical Examination 4**​.**Biochemical Profile**

**
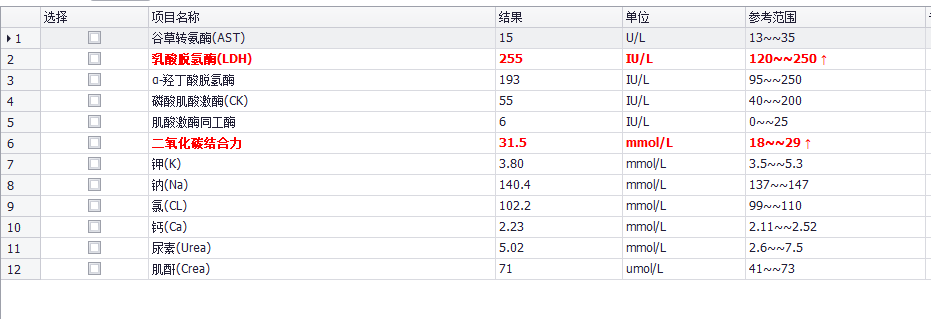
Note:**​ LDH (255 IU/L; ref: 120–250) and CO₂CP (31.5 mmol/L; ref: 18–29) were mildly elevated, suggesting nonspecific tissue damage or acid-base disturbance. Other biochemistry, including liver/renal function, electrolytes, and cardiac enzymes, remained normal. Clinical correlation and follow-up testing in 1–2 weeks are advised if indicated.

**Physical Examination 5**​.**Electrocardiogram**

**
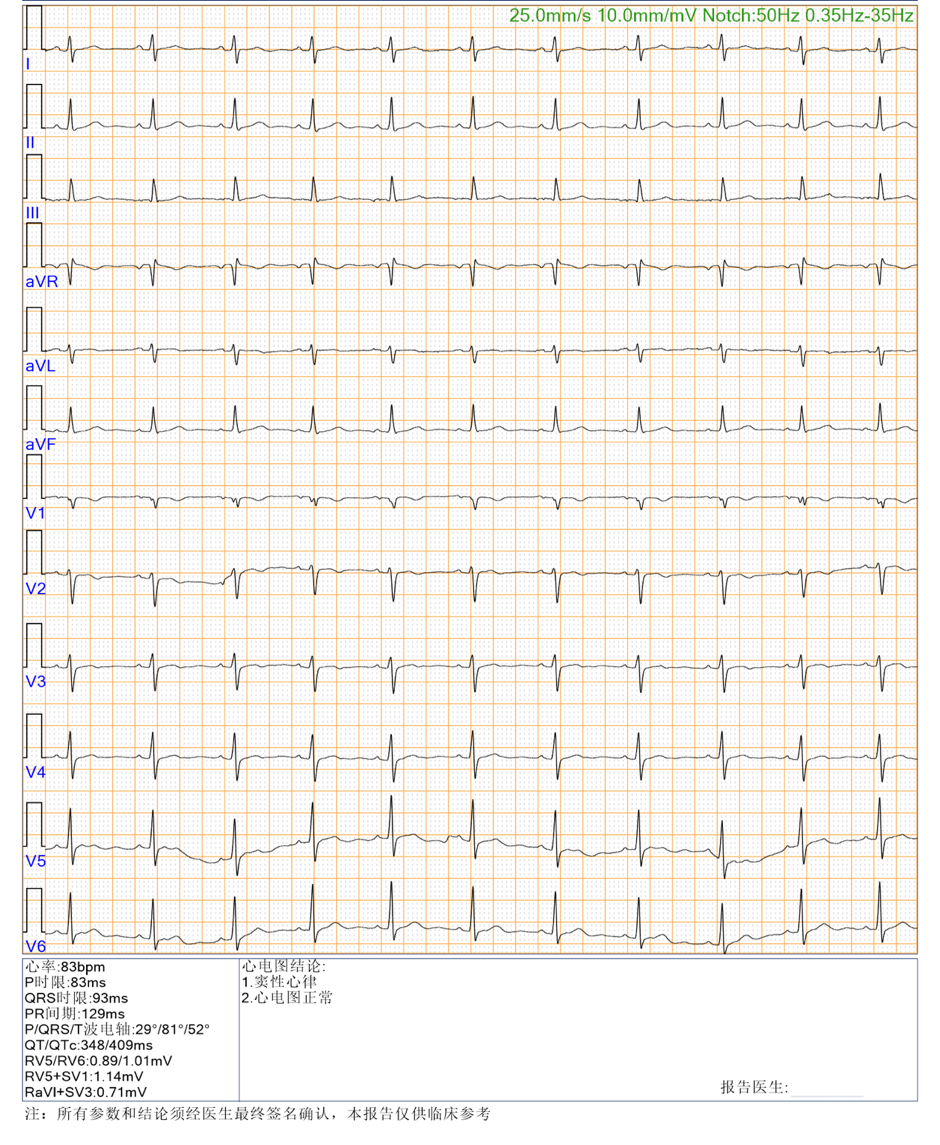
**

**
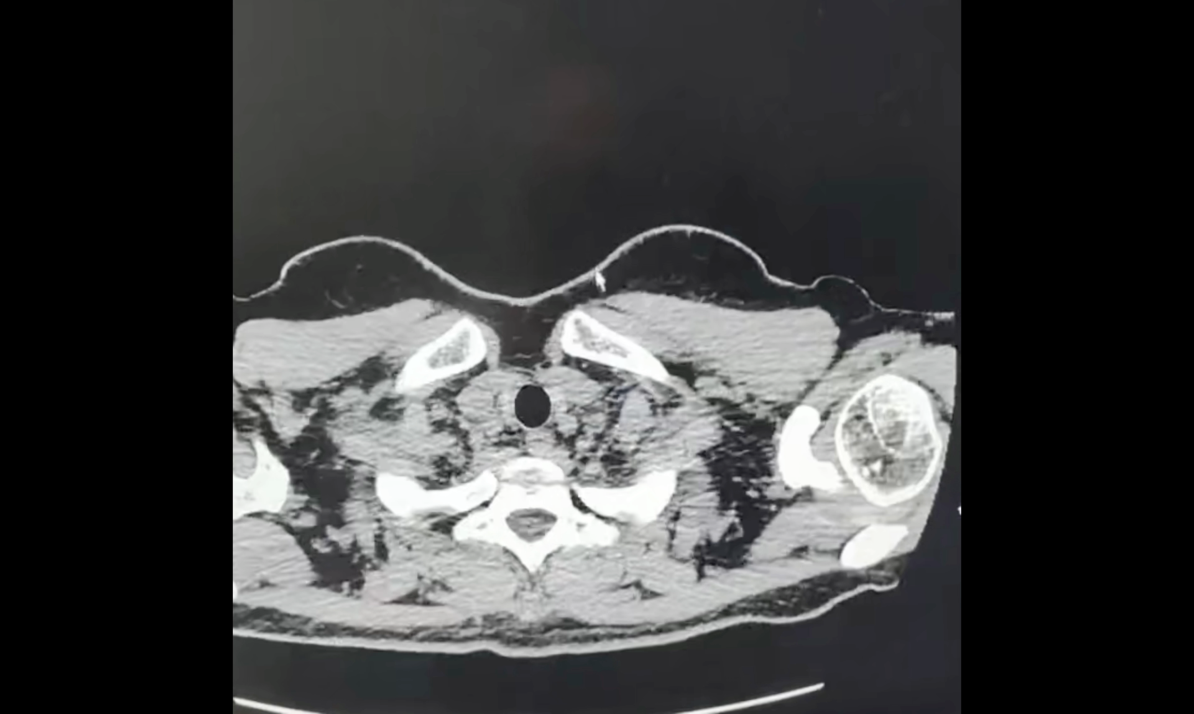
Physical**
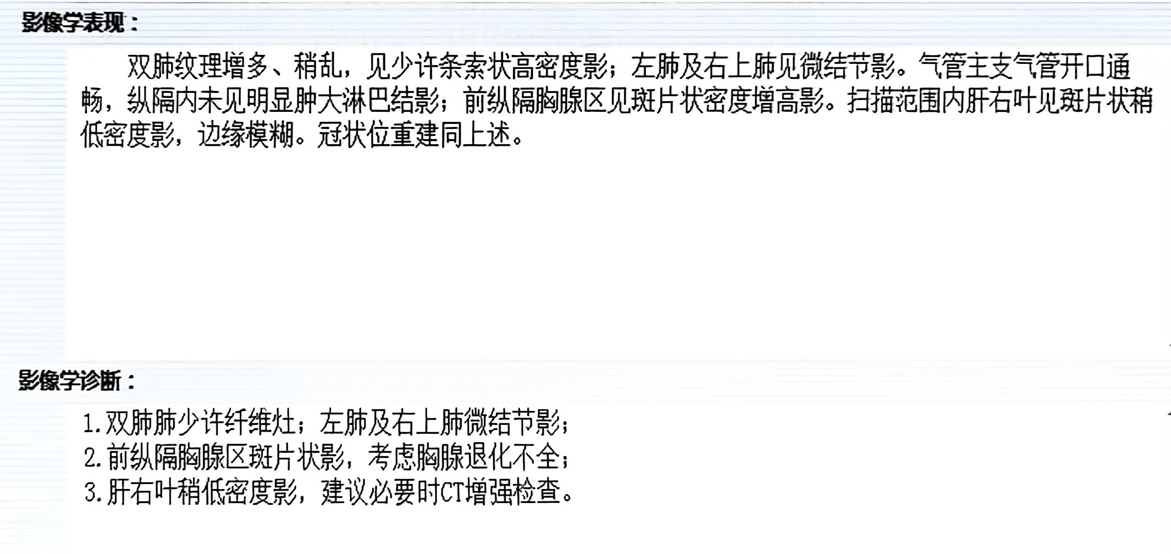
**Examination 6**​. **Screening Chest CT (video and report,** **stacked vertically)**
