# Supplementary material for: Case Report: Cardiac angiosarcoma with rib pain as the first symptom
Source: Front Oncol. 2026 Apr 13;16:1815522. doi: 10.3389/fonc.2026.1815522 (PMC13111074; doi:10.3389/fonc.2026.1815522)
Supplement: Supplementary file 1 [file SupplementaryFile1.zip › video files/Supplementary Videos .docx]

**
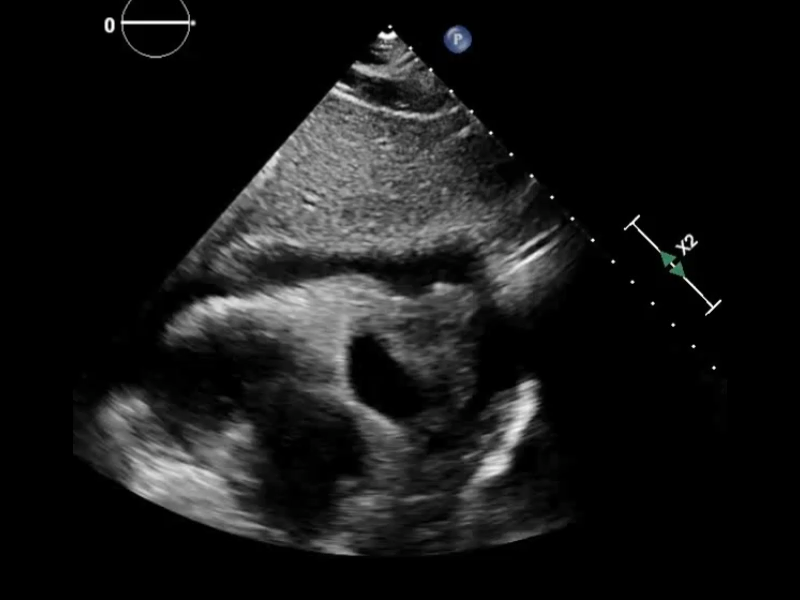
Supplementary Video 1**

**Note:** **Subcostal two-chamber view**​ showed a 43 × 30 mm lobulated, iso-echoic mass arising from the right atrial free wall, with extension into both the right atrial cavity and pericardium.

**
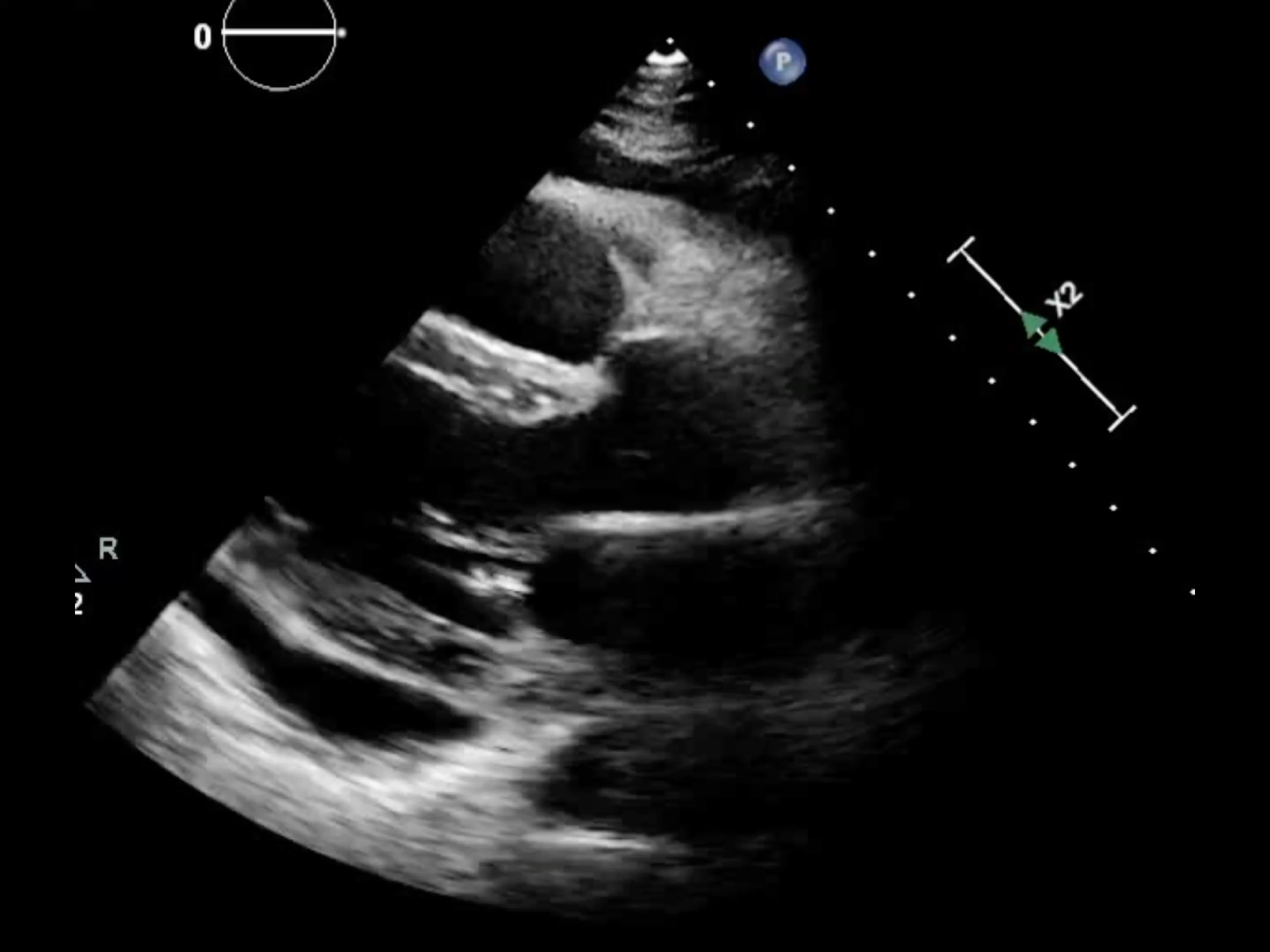
Supplementary Video 2**

**Note:** The **parasternal long-axis view**​ demonstrated pericardial effusion surrounding the right ventricular anterior wall and the left ventricular posterior wall.

**
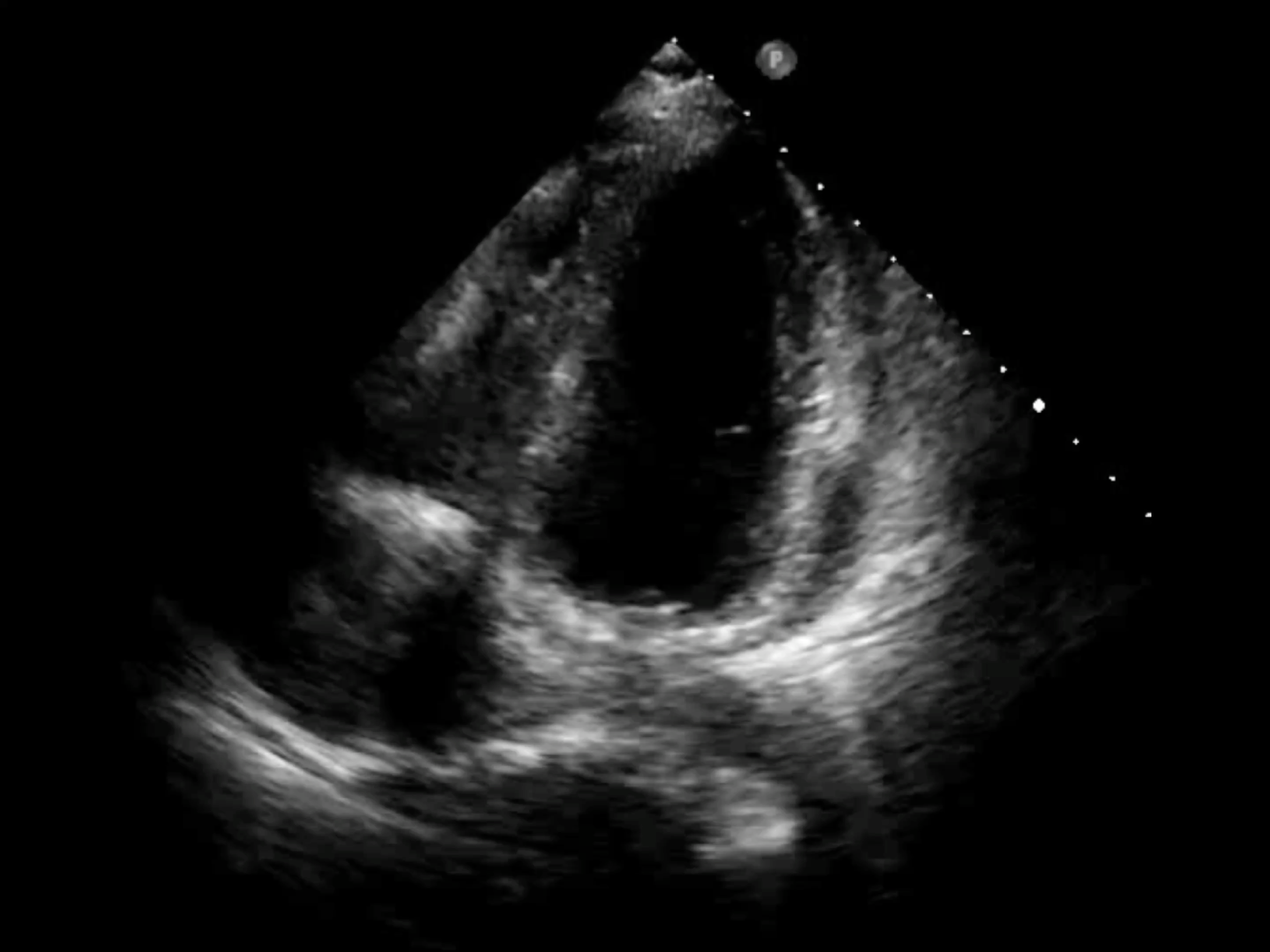
Supplementary Video 3**

**Note:** The apical four-chamber view​ demonstrated pericardial effusion along the right ventricular lateral wall, the roof of the right atrium, and the left ventricular lateral wall.

**Supplementary Video 4**

**
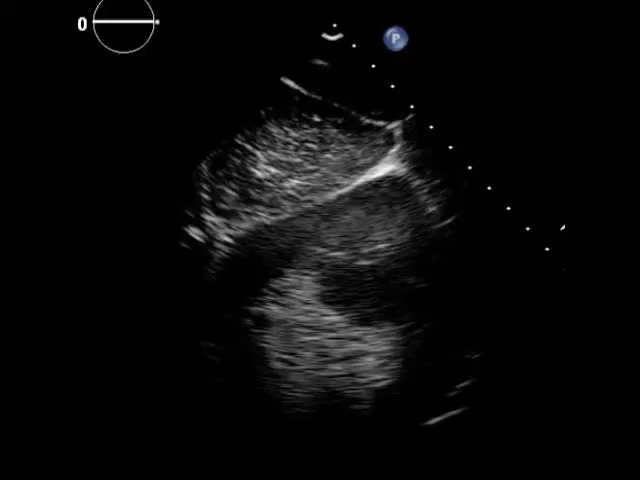
Note:** Contrast-enhanced echocardiography was performed using the subcostal two-chamber view, with the focus zone set at the right atrial level to optimize visualization of the tumor within the near field, despite the presence of an extensive pericardial effusion.
