# Supplementary material for: Case Report: Cardiac angiosarcoma with rib pain as the first symptom
Source: Front Oncol. 2026 Apr 13;16:1815522. doi: 10.3389/fonc.2026.1815522 (PMC13111074; doi:10.3389/fonc.2026.1815522)
Supplement: Supplementary file 1 [file SupplementaryFile1.zip › video files/Figure.docx]

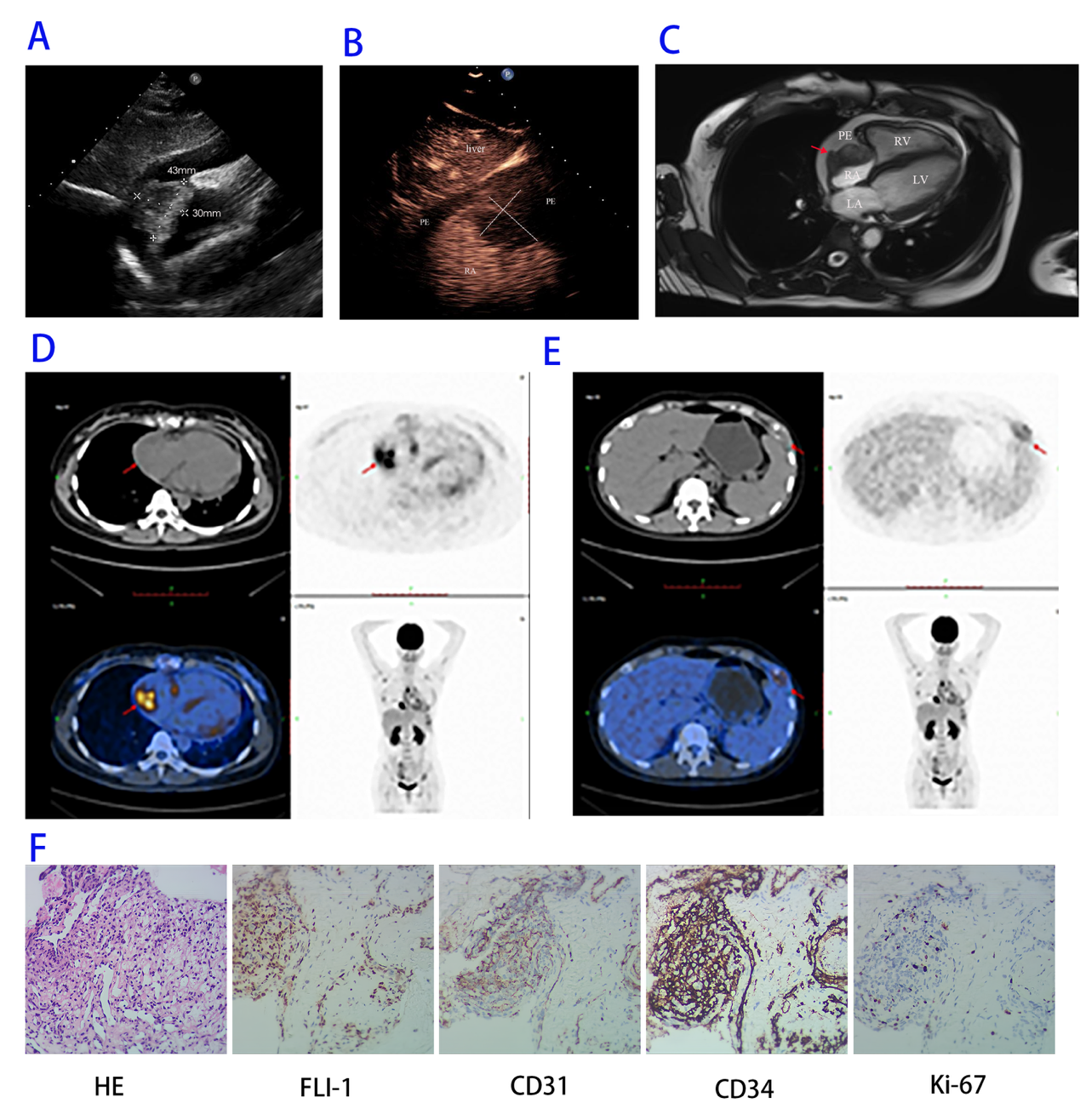


**Figure A:** Transthoracic echocardiography in the subcostal four-chamber view​ showing a lobulated, iso-echoic mass (measuring 43 × 30 mm, as indicated by calipers) originating from the right atrial free wall, with extension into both the right atrial cavity and pericardial cavity , accompanied by pericardial effusion.

**Figure B:** Contrast-enhanced echocardiography in the subcostal two-chamber view​ revealing a filling defect within the right atrium (calipers), with slight contrast enhancement within the mass.

**Figure C:** Cardiac MRI delineating the mass (arrow) originating from the right atrial free wall, exhibiting a lobulated morphology with slightly longer T1 and T2 signal intensity, accompanied by a circumferential pericardial effusion.

**Figures D-E:** PET-CT images depicting intense high metabolic activity at the right atrial mass (arrow, Figure D) and left rib lesions (arrow, Figure E).

**Figure F:** Histopathology (H&E, ×20) revealing epithelioid endothelial cells with mild-to-moderate atypia; Immunohistochemistry staining positive for FLI-1, CD31, CD34, and Ki-67 LI: 20%.
