# Supplementary figures and images for: Case Report: Cardiac angiosarcoma with rib pain as the first symptom
Source: Front Oncol. 2026 Apr 13;16:1815522. doi: 10.3389/fonc.2026.1815522 (PMC13111074; doi:10.3389/fonc.2026.1815522)

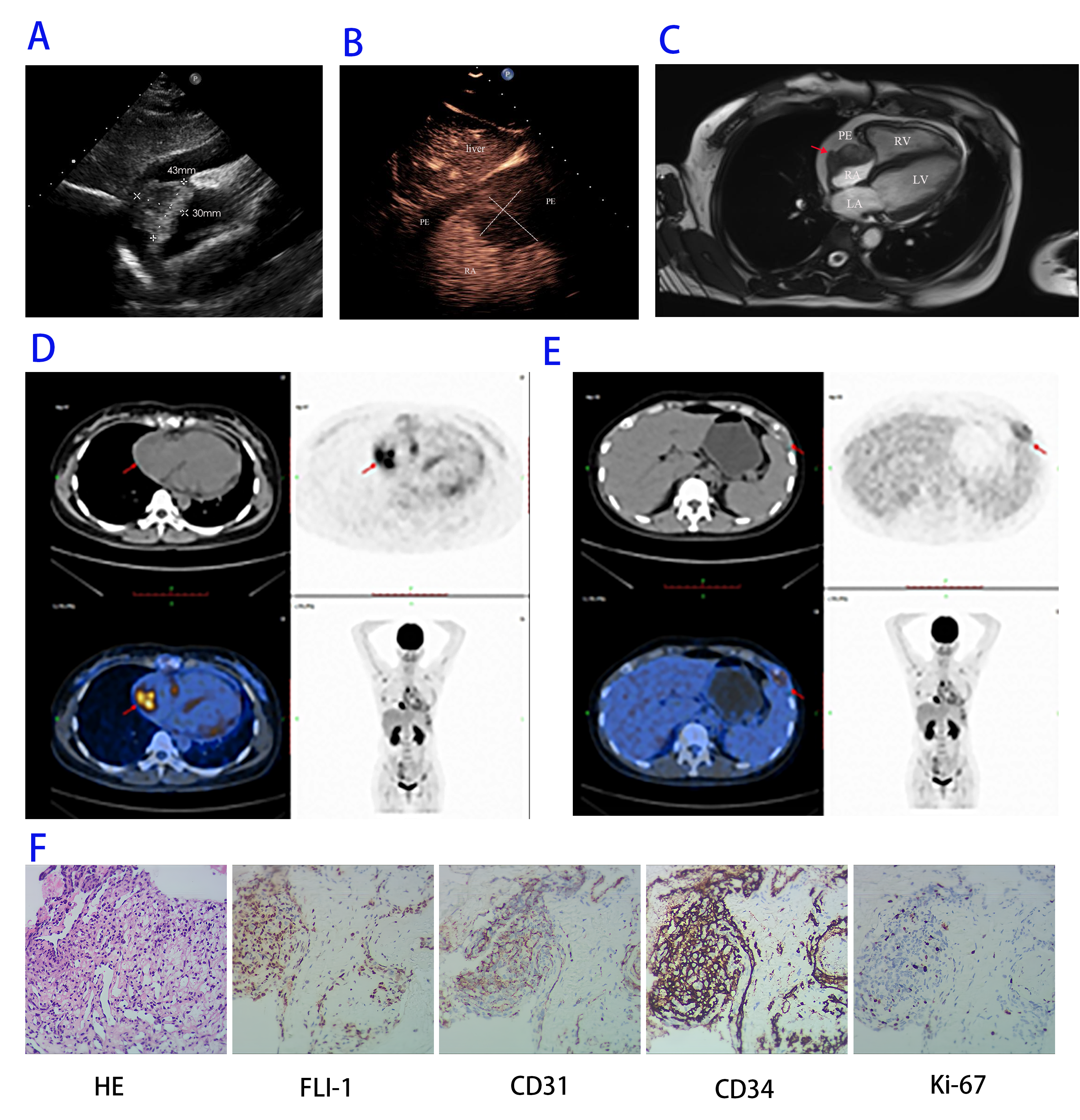

Supplement: Supplementary file 1 [file SupplementaryFile1.zip › video files/figure.tif]
